# Editorial for the Special Issue “Water Microorganisms Associated with Human Health, 2nd Edition”

**DOI:** 10.3390/microorganisms14030710

**Published:** 2026-03-22

**Authors:** Ana Machado

**Affiliations:** 1 Instituto de Ciências Biomédicas Abel Salazar (ICBAS—UP), University of Porto, Rua Jorge Viterbo Ferreira 228, 4050-313 Porto, Portugal; ammachado@icbas.up.pt; 2 CIIMAR-Interdisciplinary Centre of Marine and Environmental Research, University of Porto, Novo Edifício do Terminal de Cruzeiros do Porto de Leixões, Avenida General Norton de Matos, S/N, 4450-208 Matosinhos, Portugal

Water-associated microorganisms have re-emerged as a central public health concern, reflecting how microbial dynamics in water reflect current social, climatic, and infrastructural pressures. Hazards are now emerging across increasingly complex exposure settings, reshaping the landscape of waterborne and water-associated disease risk. Climate-driven extremes and warming aquatic environments are altering the ecological suitability and persistence of opportunistic and autochthonous pathogens in bathing, surface, and engineered waters. These shifts are transforming risk from discrete contamination events into more chronic and diffuse forms of system-mediated exposure, with clear One Health implications. At the same time, methodological advances now allow us to detect far more, and far earlier, than can yet be reliably interpreted or acted upon. The primary challenge is no longer identifying microbial signals but instead determining their relevance, comparability, and utility for prevention.

The Special Issue (SI) “Water Microorganisms Associated with Human Health” sits squarely at that intersection. Its central contribution is not an expanded catalogue of detected targets, but a clearer articulation where public health now stalls. As analytical sensitivity increases, a limiting step has become the interpretive framework, including standards, controls, comparability expectations, and exposure assumptions, that determine whether a signal can credibly support decisions. Together, the SI contributions advance a shift from detection as an endpoint toward surveillance and assessment as decision support, where uncertainty is explicit, indicators are selected for purpose, and findings are interpreted within the environmental and infrastructural systems that generate risk.

Recent developments in the field reflect a broader transition from single-organism evaluation toward integrated evidence streams that combine quantitative molecular detection, ecological context, and explicit risk framing. Wastewater-based surveillance exemplifies how assay target choice, matrix inhibition, and recovery correction become determinants of interpretability and governance rather than mere technical details (Contribution 1). Reuse-oriented monitoring is likewise moving beyond legacy bacterial indicators toward fit-to-purpose portfolios that better represent viral and spore-forming hazards under regulatory implementation constraints (Contribution 2). Environmental microbiology increasingly integrates community-level profiling and functional prediction, with attention to health-relevant traits, including resistance-associated functions in major freshwater systems (Contribution 3). Climate-sensitive risk assessment has progressed toward models that translate environmental drivers into exposure-relevant risks, including projections for naturally occurring hazards in bathing and surface waters (Contribution 4). In settings where protozoa remain a plausible and persistent concern, fine-scale ecological predictors such as rainfall and proximity to human or livestock activity are now treated as explanatory variables, demonstrating the operational value of environmental covariates (Contribution 5).

Viewed together, the SI papers make clear that one of the persistent gaps in the field is the lack of a broadly accepted framework capable of linking detection to exposure relevance, comparing findings across sites and methods, and supporting timely intervention rather than post hoc documentation. By situating methodological variability, hazard representativeness, and ecological drivers within a shared frame, the contributions redirect attention from expanding target lists towards standardization, attribution-ready designs, and decision thresholds robust to real-world variability (Contributions 1–2).

Looking ahead, the most consequential advances are likely to come from research programmes that treat interpretation as a primary object of study and explicitly design monitoring for decision use. Strengthening comparability through the routine use of recovery and inhibition diagnostics, transparent reporting conventions, and interlaboratory performance evaluations will be essential, given the meaningfully different public inferences that can arise when targets, matrices, or controls differ [1]. Equally important is the alignment of indicator strategies with risk assessment objectives. In reclaimed water applications, this includes frameworks that integrate traditional indicators with pathogen-specific markers that are more resilient or exposure-relevant, such as norovirus genogroup II and *Clostridioides difficile* spores, while evaluating rapid screening approaches for operational feasibility (Contribution 2).

The next phase of research also requires movement from static monitoring to dynamic prediction. Climate-sensitive risk assessment is increasingly feasible, but its public health value will be realized only when models are paired with monitoring triggers, management actions, and performance metrics evaluated against real-world outcomes in bathing and surface waters (Contribution 4). Evidence that ecological covariates structure protozoan occurrence further suggests that early-warning approaches must account for rapid runoff, localized persistence, and episodic amplification instead of assuming seasonal patterns (Contribution 5).

Antimicrobial resistance and virulence determinants should be treated as system outcomes that can be mitigated through engineered and catchment-scale interventions, rather than as descriptive findings appended to routine monitoring. Findings on multidrug-resistant *Enterococcus* and associated virulence genes illustrate how wastewater and receiving waters function as connected systems whose dissemination potential is shaped by treatment processes and catchment conditions (Contribution 16). Similarly, microbiome-based functional inference offers promise for prioritization under resource constraints, but it still requires rigorous validation and standardization before it can reliably inform action (Contribution 3).

Sustained attention is also warranted for neglected and opportunistic eukaryotes, particularly where environmental occurrence can be linked to plausible mechanical damage and virulence factors. Mechanistic work on *Acanthamoeba* demonstrates how cellular-level interactions and cytopathic effects connect to broader impacts on health, particularly for genotypes encountered in environmental settings but underrepresented in standard discussions (Contribution 7). Evidence of anthropogenic protists in primary drinking water sources reinforces the need for feasible monitoring and mitigation strategies that are aligned with the realities of WASH infrastructure and household exposure in resource-limited settings (Contribution 8). Equity therefore remains integral to scientific adequacy, requiring implementation research, capacity building, and co-developed monitoring strategies where preventable burden remain highest (Contribution 8).

Taken together, this Special Issue reinforces the central conclusion that detection capacity is no longer the dominant barrier to public health impact. The decisive question is whether interpretive and governance frameworks can translate measurements into robust, comparable, and effective decisions under climate change, infrastructure stress, and expanding water reuse. Progress over the next decade will depend on uncertainty-aware comparability becoming routine, on monitoring being designed around exposure contexts and decision thresholds, and on coupling surveillance to interventions and outcomes.

